# Fitness Conferred by BCR-ABL Kinase Domain Mutations Determines the Risk of Pre-Existing Resistance in Chronic Myeloid Leukemia

**DOI:** 10.1371/journal.pone.0027682

**Published:** 2011-11-28

**Authors:** Kevin Leder, Jasmine Foo, Brian Skaggs, Mercedes Gorre, Charles L. Sawyers, Franziska Michor

**Affiliations:** 1 Program for Industrial and Systems Engineering, University of Minnesota, Minneapolis, Minnesota, United States of America; 2 School of Mathematics, University of Minnesota, Minneapolis, Minnesota, United States of America; 3 Division of Rheumatology, David Geffen University of California Los Angeles School of Medicine, Los Angeles, California, United States of America; 4 Combimatrix, Irvine, California, United States of America; 5 Human Oncology and Pathogenesis Program, Memorial Sloan-Kettering Cancer Center, New York, New York, United States of America; 6 Department of Biostatistics and Computational Biology, Dana-Farber Cancer Institute, and Department of Biostatistics, Harvard School of Public Health, Boston, Massachusetts, United States of America; Duke University Medical Center, United States of America

## Abstract

Chronic myeloid leukemia (CML) is the first human malignancy to be successfully treated with a small molecule inhibitor, imatinib, targeting a mutant oncoprotein (BCR-ABL). Despite its successes, acquired resistance to imatinib leads to reduced drug efficacy and frequent progression of disease. Understanding the characteristics of pre-existing resistant cells is important for evaluating the benefits of first-line combination therapy with second generation inhibitors. However, due to limitations of assay sensitivity, determining the existence and characteristics of resistant cell clones at the start of therapy is difficult. Here we combined a mathematical modeling approach using branching processes with experimental data on the fitness changes (i.e., changes in net reproductive rate) conferred by BCR-ABL kinase domain mutations to investigate the likelihood, composition, and diversity of pre-existing resistance. Furthermore, we studied the impact of these factors on the response to tyrosine kinase inhibitors. Our approach predicts that in most patients, there is at most one resistant clone present at the time of diagnosis of their disease. Interestingly, patients are no more likely to harbor the most aggressive, pan-resistant T315I mutation than any other resistance mutation; however, T315I cells on average establish larger-sized clones at the time of diagnosis. We established that for patients diagnosed late, the relative benefit of combination therapy over monotherapy with imatinib is significant, while this benefit is modest for patients with a typically early diagnosis time. These findings, after pre-clinical validation, will have implications for the clinical management of CML: we recommend that patients with advanced-phase disease be treated with combination therapy with at least two tyrosine kinase inhibitors.

## Introduction

Chronic myeloid leukemia (CML) is caused by a reciprocal translocation between chromosomes 9 and 22 resulting in the Philadelphia chromosome which harbors the BCR-ABL oncoprotein [1], [2]. The kinase activity of BCR-ABL stimulates several signal transduction pathways that promote survival and proliferation and inhibit apoptosis [3]. The small molecule inhibitor imatinib mesylate (Gleevec, Novartis) induces a complete cytogenetic response in over 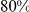 of patients with chronic phase CML [4]. However, a minority of patients in chronic phase and a substantial proportion in accelerated phase and blast crisis are either initially insensitive to imatinib therapy or lose sensitivity over time, leading to disease relapse [5], [6].

Clinical resistance to imatinib is primarily mediated by point mutations within the BCR-ABL tyrosine kinase domain [7]. To date, over 90 point mutations encoding single amino-acid substitutions have been observed (e.g. [7]–[11]). The second generation BCR-ABL inhibitors dasatinib and nilotinib are effective in most CML patients following failure of imatinib therapy. However, one potential limitation of these therapies is that their increased potency may be associated with additional side-effects [12]. In addition, none of these inhibitors have demonstrated significant activity against cells harboring the T315I resistance mutation [12]. §This limitation may be overcome by third-generation inhibitors such as ponatinib, which has recently shown promising results against T315I and is currently in late phase II trials [13].

The term ‘pre-existing resistance’ refers to the presence of drug-resistant cells prior to the start of therapy, and stands in contrast to acquired resistance which arises during the course of treatment from an apparently drug-sensitive tumor at diagnosis. The characterization of pre-existing resistance in CML is of significant clinical importance, since the likelihood and extent of resistance determines patient prognosis and treatment choices such as combination therapies and dose scheduling options. Resistant cells pre-existing at low frequencies may be the underlying cause of many cases of ‘acquired’ resistance, which are detected only after sensitive cells have been debulked by therapy. The existence and composition of pre-existing resistant clones is for many reasons difficult to ascertain [14]. It would hence be of tremendous clinical utility to develop a rational method for determining the characteristics of pre-existing resistance in CML patients.

Mathematical modeling provides a cost-effective method for studying pre-existing resistance, and many contributions have been made to elucidate the dynamics of resistance in cancer. The investigation of the dynamics of resistance mutations emerging during exponential expansion of a population was initiated by Luria and Delbrück in 1943 [15]. Their analytical results described the distribution of the number of resistant bacteria in an exponentially growing population neglecting cell death. During the last half-century, stochastic process models based on the theory suggested by Luria and Delbrück have attracted the interest of cancer researchers. However, in most situations of cancer growth, cell death cannot be neglected; thus, several authors introduced extensions of the Luria-Delbrück process that explicitly incorporate cell death (see, e.g. [16]–[18]). In pioneering work, Coldman and Goldie used stochastic process models with a differentiation hierarchy to model sensitive and resistant cancer cells, and observed that a higher rate of cell death results in a larger number of resistant cells for a given total tumor size [16]. Several other authors considered situations in which resistance emerges due to a single mutation in expanding populations using stochastic birth and death processes [17], [19]. These models were later extended to investigate scenarios in which several genetic alterations must be accumulated in a single cell for resistance to emerge [20]–[25], describing situations in which multiple alterations are required to confer resistance to a single drug, or when multiple drugs are used. Recently, evolutionary modeling has also been utilized to predict alternative therapeutic strategies capable of prolonging the clinical benefit of tyrosine kinase inhibitors against EGFR-mutant non-small cell lung cancers by delaying the development of resistance [26].

Chronic myeloid leukemia has been studied from a mathematical perspective by several authors, starting with a model of granulocytopoiesis by Fokas and colleagues in 1991 [27]. Later on, a deterministic model of the hematopoietic system in CML was considered, focusing on the dynamics of stem and progenitor cell response to imatinib [28]. These authors also calculated the probability of a single type of resistance arising due to a point mutation prior to treatment. Other contributions investigated the effects of complex phenomena such as cellular quiescence, immune system interactions, and other properties of the hematopoietic system on the dynamics of treatment response [29]–[32] Investigators also considered the emergence of cells resistant to multiple BCR-ABL inhibitors [23], [33], assuming that cells have to accumulate 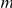 different mutations to become resistant to 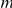 drugs. These authors calculated the probability of resistance to all drugs arising before the initiation of therapy and concluded that resistance predominantly arises prior to treatment.

These efforts have provided seminal contributions to the understanding of imatinib resistance in CML. However, several clinically important issues remain. For instance, significant fitness differences between individual resistance mutations have been observed experimentally [8]. The fitness of a particular cell type refers to the net growth or reproductive rate of each cell of that type. These fitness differences impact the genetic profile of the disease at the start of treatment, but testing limitations make it difficult to assess the characteristics of low-frequency pre-existing resistant clones in the clinic. In order to make rational clinical decisions about the use of combination therapy with second generation inhibitors, it is essential to obtain quantifiable risk assessments about the clonal diversity of resistant subpopulations at the start of treatment.

In this paper, we present the first mathematical investigation of clonal diversity within pre-existing resistant CML cells, incorporating experimental observations of the differential effect of various imatinib-resistant mutations on cellular fitness. We developed a novel mathematical model to quantify the likelihood and composition of diverse, resistant CML cells existing at the time of diagnosis. Within our framework, CML stem cells are modeled as a multi-type stochastic birth-death process, in which each cell waits a randomly distributed amount of time to give birth to a daughter cell or die. The distribution of these waiting times is dictated by the birth and death rates specified for each cell type. Once a new cell arises, it creates an independent copy of the branching process and the same reproduction and death steps are repeated. Each cell type may have distinct growth kinetics and thus proliferate and die at different rates than other types. In our model, BCR-ABL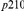-positive stem cells as well as each imatinib-resistant type are represented by cell types with distinct growth kinetics informed by experimental data [8]. During each division, BCR-ABL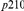-positive stem cells may give rise to an imatinib-resistant daughter cell with a small probability. To analyze the model, we first derived an estimate for the distribution of the time at which the total stem cell population reaches level 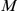. We then characterized the resistant cell population as a function of time by considering the arrival times (i.e., the time of creation) of mutated cells in conjunction with the growth and extinction dynamics of the resulting resistant clones. Lastly, we calculated the characteristics of the resistant cell population at the time of detection by convolving these estimates with the distribution of the time at which the total stem cell population reaches the detection level, 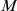.

Our results represent a significant departure from earlier work since we considered a spectrum of resistance mutations that confer a random additive change to cellular fitness. Using this model, we investigated the diversity within resistant cells present at the time of diagnosis, formulated as a stochastic hitting time – the time it takes for the stochastic population process to hit a specified cell number corresponding to diagnosis. This concept was discussed in the context of cancer modeling in [25]. We derived estimates of the risk of harboring any particular resistant cell type, the number of resistant clones, and the number of distinct resistant types present at the start of therapy. Specifically, we determined the expected number of resistance mutations present at the stochastic time of diagnosis of the disease, and studied the temporal evolution of the number of resistant types as the leukemia expands. We also investigated the frequency composition of resistant cells as a function of time and detection size. We then quantified the probability that specific mutations are present at the time of detection and the distribution of the size of these clones. Finally, we assessed the benefits of combination therapy over monotherapy in the clinical management of CML. These findings contribute to a rational understanding of pre-existing resistance in CML and may also be applicable to other cancer types treated with targeted therapy.

## Results

We utilized a stochastic multi-type branching process model to describe the emergence of multiple imatinib-resistant clones in the CML cell population prior to treatment. We considered only CML stem cells since these are the only cells capable of persisting indefinitely in the population; mutations arising in more differentiated cell types, in the absence of dedifferentiation, would be lost from the cell population by differentiation. The cell population initially consists of an expanding number of BCR-ABL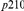-positive stem cells which may acquire various resistance mutations during cell divisions. The number of BCR-ABL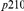-positive stem cells at time 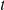 is represented by an approximation to a birth and death process with birth rate 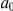, death rate 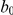, and net growth rate 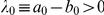. During each cell division, a resistance mutation may arise with probability 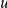. Each mutation has the potential to confer a change in the birth and/or death rate of the daughter cell; thus, each resistant type may have a different fitness, which refers to the net reproductive rate (birth rate minus death rate) of the cell. Whenever a new resistance mutation arises, a new cell type is created within the population. Denote the number of leukemic stem cells of resistant type 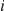 at time 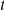 with 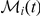. Each of these resistant types evolves according to a branching process with birth rate 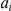, death rate 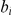, and net growth rate 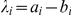. The total number of resistant stem cells is given by 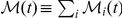. Using this evolutionary model, we then analyzed the extent and diversity of the pre-existing resistant population at the time of diagnosis of CML.

### Experimentally determined fitness parameters and mutation rate estimates

To inform the growth and death parameters of our model, we utilized data from 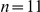 imatinib-resistant CML cell lines and a BCR-ABL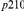 cell line [8]. Using doubling times, 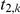, from this study, growth rates for the model were obtained utilizing the formula 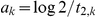. Table 1 shows the net growth rate of each cell line in the absence of therapy as well as the resistance status of each mutant [34]. Note that the T315I mutant cell line, which is resistant to all three BCR-ABL inhibitors, possesses the highest net growth rate. In addition, two other resistance mutations, Y253F and E255K, also confer growth advantages as compared to the p210 cell line; all remaining mutants have a lower net growth-rate than the p210 cells.

**Table 1:** In vitro growth rates of cells harboring resistance mutations [8].

Mutation	In vitro net growth rate ( 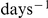 )	Std. error	Resistant to
T315I	0.271	0.0020	all
E255K	0.255	0.0043	imatinib
Y253F	0.239	0.0057	imatinib
p210	0.232	0.0041	**
E255V	0.221	0.0066	imatinib
V299L	0.203	0.0045	dasatinib
Y253H	0.202	0.0010	imatinib
M351T	0.194	0.0069	imatinib
F317L	0.188	0.0134	imatinib, dasatinib
T315A	0.187	0.0057	dasatinib
F317V	0.171	0.0075	dasatinib
L248R	0.144	0.0061	imatinib, dasatinib

The table displays the type of resistance mutation (ordered in terms of decreasing growth rate), net growth rate (mean and standard error), and sensitivity to the tyrosine kinase inhibitors imatinib, dasatinib, and nilotinib. p210 refers to the drug-sensitive BCR-ABL-positive cell type.

We utilized the *in vitro* data outlined above to estimate the birth and death rates, 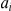 and 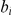, of these resistant types *in vivo*. We obtained estimates of the *in vivo* birth and death rates of BCR-ABL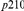 leukemic stem cells, 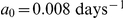 and 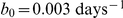, as reported in [28], leading to a net growth rate of 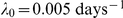. We then determined a constant conversion factor relating *in vitro* net growth rate estimates of BCR-ABL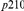 leukemic stem cells to their *in vivo* net growth rate, and applied this conversion factor to the data in Table 1 to obtain estimates of the *in vivo* net growth rates of resistant cells. The estimates of *in vivo* growth and death rates are specified in Table 2. The point mutation rate, 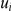, was considered to be constant across the eleven mutations, such that during each cell replication, all resistance mutations are equally likely to arise. This assumption can be relaxed by considering differing mutation rates; however, in the absence of experimental data that can be used to inform the magnitude of mutation rate differences, we have chosen to consider constant mutation rates for all eleven mutants. An estimate of 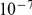 was used chosen for the mutation rate (although in a later section we also analyze the sensitivity of model predictions to perturbations in the mutation rate). This estimate was chosen since the baseline mutation rate of human cells has been characterized as approximately 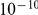 per base per cell division [35], while the mutation rate of malignant lymphoid cells was observed to be up to 1000-fold larger than this baseline value [36]. Although this latter observation was obtained from lymphoid cells, it is consistent with experimental evidence for CML cells, whose mutation rates were observed to be increased by several hundred-fold as compared to parental cells not carrying the BCR-ABL oncogene [37]. In this model we have neglected this possibility of back mutation from a resistant mutant to sensitive phenotype, since the chance that a cell acquires a mutation that reinstates the original wild type state is exceedingly small. This assumption is a very well-established modeling choice in population genetics, where it is called the infinite-site assumption.

**Table 2:** In vivo growth rates of cells harboring resistance mutations.

Mutation	In vivo growth rate ( 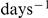 )	Resistant to
T315I	0.0088	all
E255K	0.0085	imatinib
Y253F	0.0082	imatinib
p210	0.008	**
E255V	0.0078	imatinib
V299L	0.0074	dasatinib
Y253H	0.0074	imatinib
M351T	0.0072	imatinib
F317L	0.0071	imatinib, dasatinib
T315A	0.0070	dasatinib
F317V	0.0067	dasatinib
L248R	0.0061	imatinib, dasatinib

The table shows the estimated *in vivo* birth rates for the eleven BCR-ABL resistant mutants (ordered in terms of decreasing growth rate) and the BCR-ABL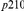 cells. The death rate for all cell types is 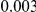 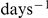.

### Distribution of the detection time

The time of diagnosis of the disease is defined as the time at which the total number of CML stem cells reaches a threshold detection size, 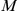. This quantity was estimated to be approximately 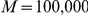 in chronic phase patients [38], [39]. We determined characteristics of resistant clones for this estimate of 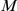, as well as for larger 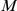 in order to quantify the effect of later detection on the dynamics of resistance. Since CML stem cells evolve according to a stochastic process, the time at which the total cell number reaches size 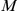 is a random quantity, 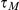. We derived an approximate form for the probability density function of this random time, given by
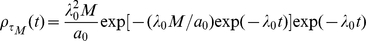
This density function was determined by considering the asymptotic behavior of the single type birth-death process representing the BCR-ABL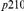-positive CML stem cells. In the parameter regime of interest (defined by point mutation rates per cell division and growth kinetics of the resistant types), the random time that the BCR-ABL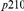-positive CML cell number hits a threshold size 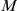 represents a good approximation of the time at which the total CML stem cell number hits size 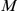. We also obtained an expression for the mean of the detection time, given by
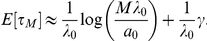
where 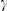 is the Euler-Mascheroni constant. The median of this distribution is given by
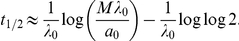
Derivations of these formulae as well as an expression for the mode of this distribution is provided in Material S1.

Figure S1 displays the density function of the detection time for three different example abundances of leukemic stem cells at diagnosis. As expected, a larger detection size results in a shift of this density to the right, indicating later detection times. For the baseline detection size estimate of 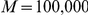 leukemic stem cells, diagnosis is expected to occur around 6–7 years after cancer initiation; this time frame is consistent with data from Hiroshima bomb survivors [40]. In the following analyses, the density function plays an important role in ascertaining the characteristics of resistant cells at the detection time.

### The number of distinct resistant types present at diagnosis

A straightforward method of characterizing biodiversity within a population is to report the number of individual distinct types or ‘species’; this measure of diversity is often referred to as *species richness* in ecology [41]. Using the density function 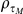 calculated previously, we next studied the number of distinct cell types, 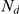, present at the time of diagnosis. Let 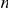 be the total number of resistant types. Then the mean number of distinct resistant types present at diagnosis is given by
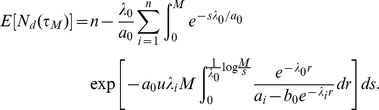
We also derived the probability mass function for the number of resistant types present at the time of detection. For each 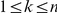, define 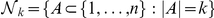 (i.e. all possible subsets of resistant types with cardinality 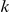). Then the probability of having 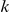 distinct types present at diagnosis is given by
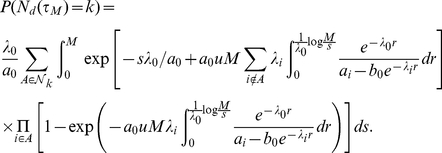
Derivations of these expressions are provided in Material S1.

**Figure 1:**
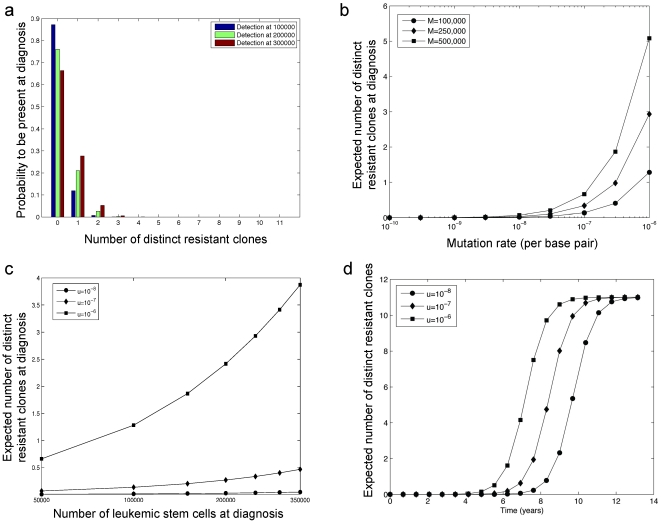
Existence and composition of pre-existing resistance. (a) The panel shows the distribution of the number of distinct resistant clones at the time of diagnosis. There are 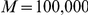 (blue), 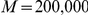 (green), or 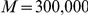 (red) leukemic stem cells present at the time of diagnosis. The per base pair mutation rate is 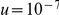. (b) The panel displays the expected number of resistant cell types as a function of the detection size, 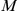, for varying mutation rates. (c) The expected number of resistant types at diagnosis is shown as a function of the mutation rate 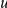, for varying numbers of leukemic stem cells at diagnosis, 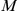. (d) The expected number of resistant cell types is displayed as a function of time 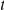 in years, for varying mutation rates 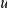.

Using these approximations, we examined the distribution of the number of distinct resistant leukemic cell types present at normal and late detection times (Fig. 1a). For the baseline estimate of 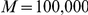 leukemic stem cells at the time of diagnosis of the disease, almost all patients harbor zero or one resistant clone at diagnosis. The probability of harboring no resistant cells at diagnosis is approximately 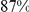, while the probability of having exactly one resistant cell type is approximately 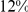 if the mutation rate is 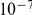 per base per cell division. We also tested for the sensitivity of the results to the estimate of the mutation rates (see Fig. 1b). When 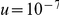, the chance that a newly diagnosed patient harbors two or more resistant cell types is approximately 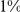. If the disease is detected at later times, then the probability mass function shifts to the right and slightly more resistant types are present. However, for all detection sizes tested, the probability of greater than three resistant types present at the time of diagnosis is negligibly small. Although it is biologically possible to acquire more than one mutation in a single cell division, this event has negligible probability given the scale of the mutation rate and detection size. Thus in the these analyses we do not explicitly discuss cell populations with multiple resistance mutations.

These results provide useful quantitative guidelines for the clinical management of CML: patients diagnosed with a disease burden of about 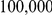 leukemic stem cells have an approximately 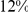 chance of harboring one imatinib-resistant cell type. Patients diagnosed later (e.g. with 200,000 leukemic stem cells) have a significantly greater chance of harboring one resistant cell type (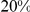) or even two distinct types (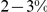). Thus, the disease burden at the start of treatment is an important indicator of the likelihood of harboring one or multiple resistant types at the start of treatment, and may be useful in making first-line treatment choices for individual patients. In particular, the total cost and potential toxicity of administering a second-generation inhibitor (either in lieu of or in addition to imatinib) should be considered along with the chance of harboring one or multiple resistant cell types.

We also considered the effects of increasing detection size and mutation rates on the expected number of distinct types of resistant cells present at diagnosis. Figure 1b displays the expected number of resistant types, 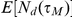, as a function of the detection size, 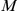, for various values of the mutation rate, 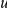. In Figure 1c, we show 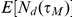 as a function of the mutation rate for various choices of 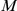. Increasing detection size and mutation rate both lead to a larger expected number of resistant cell types present at diagnosis. These studies could be extended to include mutant-specific mutation rates, which will be the topic of future contributions.

We then studied the evolution of this diversity measure (i.e., the number of distinct resistant clones) over time as the disease progresses. We found that the expected number of resistant types as a function of time can be written as
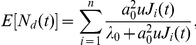
where
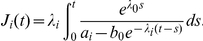
Similarly, the variance can be written as
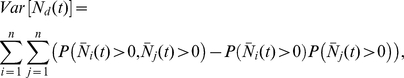
where 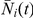 represents the number of extant mutations that have been created by time 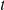. The derivations of these expressions are provided in Material S1. Figure 1d displays the expected number of resistant cell types as a function of time, 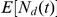, for three values of the mutation rate. For the baseline estimate of 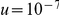 per base per cell division, the expected number of types increases substantially after six years of disease evolution. Intense diversification of the resistant cell population occurs at slightly different times for the other mutation rate estimates, but remains close to the 6–7 year range. Thus, a delay in detection has two negative consequences: it increases the likelihood of pre-existing resistant cells, and it enhances the diversity of resistant cells.

### The number of pre-existing resistant cells

We then studied the number of resistant cells of each type as a function of time, as well as at the time of diagnosis; quantifying the abundance of each type is important since small populations of resistant cells may become extinct due to stochastic fluctuations. Thus, resistant types that routinely establish large clones are likely more prevalent in the clinic.

The expected population size of each resistant cell type at time 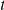 is given by
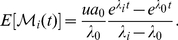
(1)Accurate analytical estimates of the expected number of resistant cells at detection time 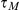 were obtained for those resistant cell types whose net growth rate was less than that of the p210 cells,
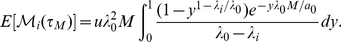
These formulae are derived in Material S1.

**Figure 2:**
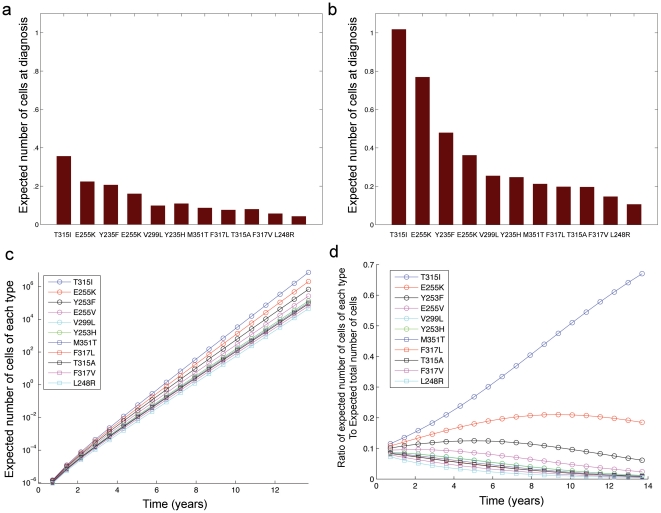
The frequency of pre-existing resistant cells. (a) The panel displays the expected number of resistant cells of each type at diagnosis for 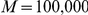 leukemic stem cells. (b) The expected number of resistant cells of each type at diagnosis for 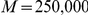 leukemic stem cells is shown. (c) The panel displays the time evolution of the average number of cells of each type. (d) The ratio of the expected number of cells of each type to the expected total resistant cell number as a function of time. The mutation rate is 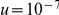 per base per cell division for all panels.

Figure 2 displays the expected numbers of various resistant types both prior to and at the detection time. Panels a and b show the expected number of cells of each type for 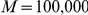 and 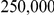 leukemic stem cells at detection time, respectively. Note that for larger detection sizes, the total number of resistant cells increases and the dominance of the T315I mutation over other types is even more pronounced. This effect is further investigated in Figure 2c and d. Panel c shows the time evolution of the average population size of each type as calculated from equation (1); six to seven years after cancer initiation, the expected number of resistant cells is on the order of one cell out of a total population of approximately 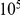 leukemic stem cells; thus only an experimental technique with sensitivity greater than 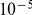 stem cells would detect resistant leukemic stem cells at this frequency. In addition, the histogram in Figure 2d displays the ratio of the average number of each type to the expected total number of resistant cells as a function of time. At early times (e.g., 0 to 2 years after cancer initiation), the mutant types on average establish similarly-sized clones. However, as time passes, the fitter types such as T315I- and E255K-positive cells give rise to much larger clones as compared to the other types.

**Figure 3:**
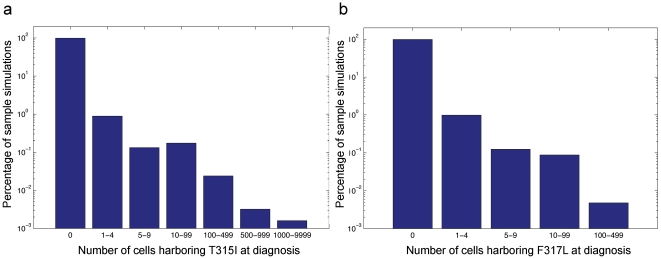
The frequency of T315I and F317L mutations at diagnosis. The figure shows the distribution of the number of T315I-positive (a) and F317L-positive (b) cells in the population at detection time. Parameters are 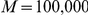 and 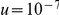, and simulations are run for 100,000 samples.

Two of the most important resistant cell types are those harboring the T315I and F317L mutations. The abundance of these resistant cell types are of particular clinical importance since the T315I mutation confers resistance to all three BCR-ABL inhibitors, while the F317L mutation confers resistance to two of them. We therefore studied the distribution of these two resistance mutations at detection time. Figure 3 displays the distributions of resistant cells for a series of sample simulations. While both distributions have the most mass accumulated at the origin, large T315I clones (i.e., 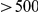 cells) arise with much greater frequency than large F317L clones. In addition, medium-sized T315I clones (i.e., 100–500 cells) emerge in roughly 2% of samples, while F317L clones of similar size arise in only 0.5% of cases. When investigating the other resistance mutations, we found that T315I-positive cells are more likely to create a large clone at detection than any other resistant cell type. This effect is amplified in tumors that are detected later, i.e. when the CML stem cell pool is larger. Figure S3 provides analogous histograms for the remaining 9 mutant cell types.

The histograms discussed above were generated via repeated independent stochastic simulations of the branching process model. Each simulation run was initiated with a single BCR-ABL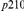-positive stem cell which waits an exponential amount of time until the first event occurs, which is either a cell division or a cell death event. This random waiting time is exponentially distributed with the mean given by the inverse of the sum of the birth rate, 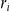, and death rate, 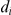, for this cell type 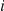. Once an event occurs, a choice is made for the cell to either divide or die; the cell divides with probability 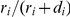 or otherwise dies. During each division event, there is a small probability 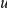 that a sensitive cell mutates to produce a resistant daughter cell (belonging to one of the 11 resistant types). This resistant cell then creates an independent clone governed by a branching process with the corresponding birth and death rates, as provided in Table 2. This process is repeated until the total population of cells reaches the detection size 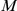, at which point we recorded the number of cells of each resistant type. Tabulating this information over repeated independent trials led to the histograms displayed in Figure 3 and Figure S3.

### The risk of pre-existing resistance

We then investigated the likelihood that a patient harbors any specific resistance mutation at the time of detection. Using our stochastic model, we derived the probability that there are no resistant cells of type 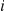 at detection time as
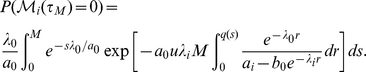
The likelihood of any resistant cell type existing in the population at detection time then easily follows as complement of this quantity. The details of the derivation are provided in Material S1.

**Figure 4:**
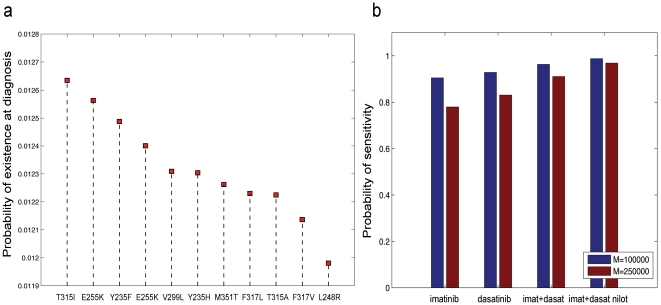
Sensitivity to therapy. (a) The panel displays the probability that there exists at least one cell with any specified mutation in the CML stem cell population at detection time. Parameters are 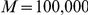 and 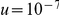. (b) The probability that the leukemic stem cell population is free of mutants conferring resistance to imatinib, dasatinib, imatinib plus dasatinib, and all three drugs at detection time. Parameters are 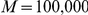 (red), 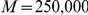 (blue), and 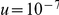.

Figure 4a displays the probability that each of the eleven resistant cell types is present at the time of diagnosis with 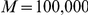 leukemic stem cells. The likelihood of any cell type existing is nearly identical (about 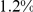) for all of the eleven types. Recall that the majority of patients harbor at most one type of resistance mutation at detection (see Fig. 1). This observation, in conjunction with the discovery that all mutations are equally likely to be present, led us to conclude that patients are equally likely to harbor T315I-positive cells at diagnosis as any other resistant cell types. However, as we observed in the previous section, T315I-positive cells are more likely to establish larger cell clones, making this mutation more clinically apparent even though it is not more frequent at the time of diagnosis. In other words, when present, T315I-positive cells are more likely to establish larger clones at detection time than other types; this may result in an increased *perceived* frequency of this mutation.

### Sensitivity of newly diagnosed CML patients to tyrosine kinase inhibitors

We then investigated the probability that a newly diagnosed CML patient responds to one or a combination of the BCR-ABL inhibitors imatinib, dasatinib, and nilotinib. In this context, sensitivity to a particular inhibitor is defined as the absence of cells containing any of the eleven mutations which confer resistance to that drug; this analysis can be performed for eleven mutations only since no information on fitness parameters is available for other resistance mutations. Figure 4b displays the probability that a patient is sensitive to imatinib, dasatinib, the combination of imatinib and dasatinib, and a three-drug combination. This analysis was performed considering two different threshold numbers of CML stem cells at diagnosis; as expected, a larger detection size leads to a higher risk of resistance. Note that even if two drugs cannot be administered at the same time for reasons of toxicity, within our framework, alternating therapy still represents a significant improvement over therapy with a single inhibitor.

Interestingly, for patients in which the disease was diagnosed when there were 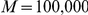 leukemic stem cells, the benefit of combination therapy over monotherapy with imatinib or dasatinib is quite small; the probability of sensitivity to imatinib or dasatinib is in the range of 90–95%, while the probability of sensitivity to imatinib and dasatinib or a three-drug combination therapy is approximately 95–98%. This observation is due to the fact that at this detection size, it is very unlikely that multiple types of resistant cells co-exist in the patient. However, for a larger disease burden at diagnosis, a significant benefit arises from utilizing combination therapies over imatinib or dasatinib monotherapy, since there is a pronounced risk of harboring more than one type of resistant cell. In this case, the probability of sensitivity to imatinib is less than 80%, while the probability of sensitivity to a three-drug combination is over 95%. Thus, the increase in effectiveness of combination therapy over monotherapy with imatinib or dasatinib is much more pronounced for patients whose disease is diagnosed later as compared to early chronic phase patients. Therefore, for patients in advanced stages of the disease, it is preferential to administer combination therapy of at least two BCR-ABL tyrosine kinase inhibitors, such as imatinib and nilotinib.

### Robustness analysis

We also tested the robustness of the results to reasonable perturbations which may account for errors in the experiment data – i.e. the growth kinetics of the eleven resistant cell types – or in our model assumptions. One key assumption of our framework is that the ratio of net growth rates of resistant and BCR-ABL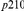-positive cell lines is preserved in patients. To determine the amount of robustness of our findings to errors in this assumption, we perturbed the *in vivo* growth rate estimates with scaled uniform random variates representing 20% noise simultaneously for all 11 mutants, and observed that our results were extremely robust to this level of noise. The level of 20% noise was chosen to be in excess of the experimental noise level in the measurement of *in vitro* growth kinetics ([8], see Table 1 for the experimental standard error). Table S1 shows the minimum and maximum probability of sensitivity to each drug combination, obtained after 100 sample simulations of the perturbed system. In each sample simulation, a perturbed growth rate was drawn at random for each mutant, and the probability of sensitivity to each drug combination was computed. This numerical experiment was conducted for two different values of the detection size. We observed that the range of the resulting probabilities for each drug combination, as reported via the minimum and maximum observed, remarkably deviated by less than one percent from the probabilities obtained from the unperturbed growth rates. To demonstrate visually how robust the results were, Figure S2a shows the resulting probabilities for one sample simulation; these results should be compared to Figure 4b.

Another modeling choice of uncertain biological underpinning relates to whether the differences in cellular fitness are due to differences in birth or death rates. Since the BCR-ABL oncogene modulates the proliferative potential of cells [42], we hypothesized that the BCR-ABL kinase domain resistance mutations likely also affect growth more than death. Therefore, we first considered differences in cellular fitness to be due to differences in birth rates, so that the death rate remains unchanged among resistant cell types (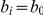 for 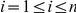). We then investigated the robustness of our results by comparing this situation to the one in which differences in fitness are attributed to differential death rates (see Figure S2b), and obtained results which deviated less than one percent from the original results shown in Figure 4b.

## Discussion

In this paper, we have developed a stochastic mathematical model for the evolution of multiple types of resistance mutations to BCR-ABL tyrosine kinase inhibitors (TKIs) within an expanding population of leukemic stem cells before the initiation of therapy. Using experimental studies of the fitness values of cells harboring different resistance mutations [8], we investigated the likelihood, composition, and diversity of pre-existing resistance as well as the impact of these factors on the response to targeted therapies.

Our study led to several conclusions regarding pre-existing TKI resistance in CML patients:

- The majority of patients harbors at most a single resistant clone at detection; the likelihood of more than one resistant cell type being present at detection is small unless the disease is detected in advanced stages, i.e. with a large pool of CML stem cells at diagnosis.
- The likelihood of harboring the T315I mutation at detection is similar to the likelihood of harboring any other resistant mutation. However, when present, T315I-positive cells are more likely to establish more clinically apparent, large-sized clones at detection time than other types; this may result in an increased *perceived* frequency of this mutation.
- Delayed detection increases the risk of pre-existing resistance and also enhances the diversity of the resistant population at diagnosis.
- The overall benefit of combination therapy over monotherapy with imatinib or dasatinib is minor for patients with a small disease burden at detection, i.e. for those who are diagnosed early. However, this benefit is significantly more pronounced for patients whose disease is diagnosed late. Thus, within our framework, combination therapy is beneficial for patients diagnosed with a large disease burden.

Our combined theoretical and experimental approach predicts that 6–7 years after cancer initiation, the expected number of resistant cells is approximately 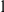 resistant leukemic stem cell out of a total population of 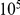 leukemic stem cells; thus an experimental technique with sensitivity capable of identifying one resistant leukemic stem cell out of 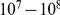 leukemic cells is needed to detect resistant cells of this frequency. In addition, at early times (e.g. 0 to 2 years after cancer initiation), all resistant cell types on average establish similarly-sized clones. However, as time increases, the fitter types – such as the T315I and E255K mutants – give rise to much larger clones as compared to other mutations. These findings emphasize the necessity to quantify the number of leukemic stem cells over time since this disease burden determines the risk of relapse due to the evolution of TKI resistance.

In this study, we initially assumed that the ratio between net growth rates of BCR-ABL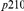-positive and resistant leukemic stem cells *in vitro* is preserved *in vivo*, and that differences in cellular fitness between resistant types are due to differences in birth rates instead of death rates. To investigate the impact of these assumptions, we studied the robustness of our results to variation in resistant cell growth and death rates. For example, we investigated the scenario in which the difference in net growth rate among resistant mutants is due to variation in death rates instead of birth rates. We found that our results (i.e. the characteristics and diversity of the resistant population at detection time) are very robust to this change. For example, Figure S2b shows the probability that a patient is sensitive to imatinib, dasatinib, the combination of imatinib and dasatinib, and a three-drug combination when the fitness difference is attributed to death rates. These results are very similar to the analogous Figure 4b where the fitness difference is attributed to growth rates. More generally, our model demonstrated that the scale and parameters of this system (i.e. mutation rates, detection size, etc) imply that resistant mutants usually arise late in the time-scale and close to the time of detection. Furthermore, the differences in growth rates among these mutants are not large enough to enable the selection of some types over others, given the short time period between the creation of the mutants and the detection time. Thus, even though fitness differences exist between these mutants, detection occurs so close in time to mutant creation that there is an approximately equal likelihood of finding each one in the population at detection. Our key findings hold for all reasonable perturbations of our *in vivo* growth rate estimates. To demonstrate this finding, we perturbed the *in vivo* growth rate estimates with scaled uniform random variates representing 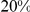 noise and recalculated the probabilities of sensitivity to combination and monotherapy. Figure S2a displays these probabilities for one sample perturbation of growth rates, and Table S1 shows the minimum and maximum values for each probability, obtained from 100 trials of randomized perturbation. These findings support the conclusion that our results are robust to substantial perturbations in the *in vivo* growth rate estimates.

We have focused on resistant cells arising prior to the start of therapy since it is difficult to detect and characterize low-frequency pre-existing resistance in the clinic. This analysis could be extended to study the diversity and composition of resistant cells arising during treatment if experimental data on fitness parameters of different cell types during therapy was available. Specifically, we could utilize mathematical modeling to quantify the impact of drug combinations and scheduling choices on the evolution of resistance. Further, data on additional mutants not considered in this study is necessary to obtain an accurate picture of the dynamics of these resistance mutations in CML patients. Our model can also be utilized to study the scenario in which the rate of the creation of resistant cells (i.e. mutation rates) vary amongst the different types. If these mutation rates are experimentally found to vary significantly across several orders of magnitude, such findings would impact the clinical predictions of our model. Finally, we have only considered cell-autonomous factors in this mathematical framework and have neglected more complex factors such as interactions with the microenvironment and immune system; this choice was made since no quantitative data on the interactions between these factors and CML stem cells are available *in vivo*. An extension of mathematical approaches to resistance in CML including such factors is an important goal of the field.

The type of analysis presented in this paper can aid in the design of therapy scheduling to delay or prevent the evolution of resistance. Our methodology can be applied to studying other cancer types and therapies which are susceptible to diverse resistance mutations, as in the case of CML. We have derived simple analytical estimates of the number of resistant types present at detection, the probability that any given resistant cell type is present at diagnosis, and the number of cells of each resistant type. This interdisciplinary approach provides a useful tool for researchers to predict the likelihood and characteristics of diverse pre-existing populations as well as the potential benefits of various therapies using only growth kinetics data as input.

## Methods

Please refer to Material S1 for detailed explanation of all formulas in the text as well as simulation details.

## Supporting Information

Figure S1 **Time of detection of disease.** The figure shows the probability density function of the detection time, or the time that the CML stem cell population hits size M, for M = 100, 000, 250, 000 and 500, 000. Since the mutant population does not represent a significant portion of the population at detection, this distribution is closely approximated by considering the time at which the number of drug-sensitive CML stem cells reaches M.
(TIFF)

Figure S2 **Robustness to growth rate perturbations.** a) Probability of sensitivity to mono- and combination therapies when the resistant mutant birth rates are perturbed by a multiplicative random factor (1+0.2·U [−1, 1]), for one representative sample (see Table S1 for comprehensive robustness statistics). b) Probability of sensitivity to mono- and combination therapies when the fitness differences between mutants are attributed to variation in death rates instead of birth rates. In both panels, probabilities are shown for detection sizes of 100,000 and 250,000 cells.
(TIFF)

Figure S3 **The frequency of CML resistance mutations at diagnosis.** The figure shows the distribution of the number of Y253H-positive (a), Y253F-positive (b), V299L- positive (c), T315A-positive (d), M351T-positive (e), L248R-positive (f), F317V-positive (g), E255V-positive (h), and E255K-positive (i) cells in the population at detection time. Parameters are M = 100, 000 and u = 10−7, and simulations are run for 100,000 samples.
(TIFF)

Table S1 **Robustness properties.**
(PDF)

Material S1 **Supplementary Material.**
(PDF)
