# Supplementary figures and images for: Fitness Conferred by BCR-ABL Kinase Domain Mutations Determines the Risk of Pre-Existing Resistance in Chronic Myeloid Leukemia
Source: PLoS One. 2011 Nov 28;6(11):e27682. doi: 10.1371/journal.pone.0027682 (PMC3225363; doi:10.1371/journal.pone.0027682)

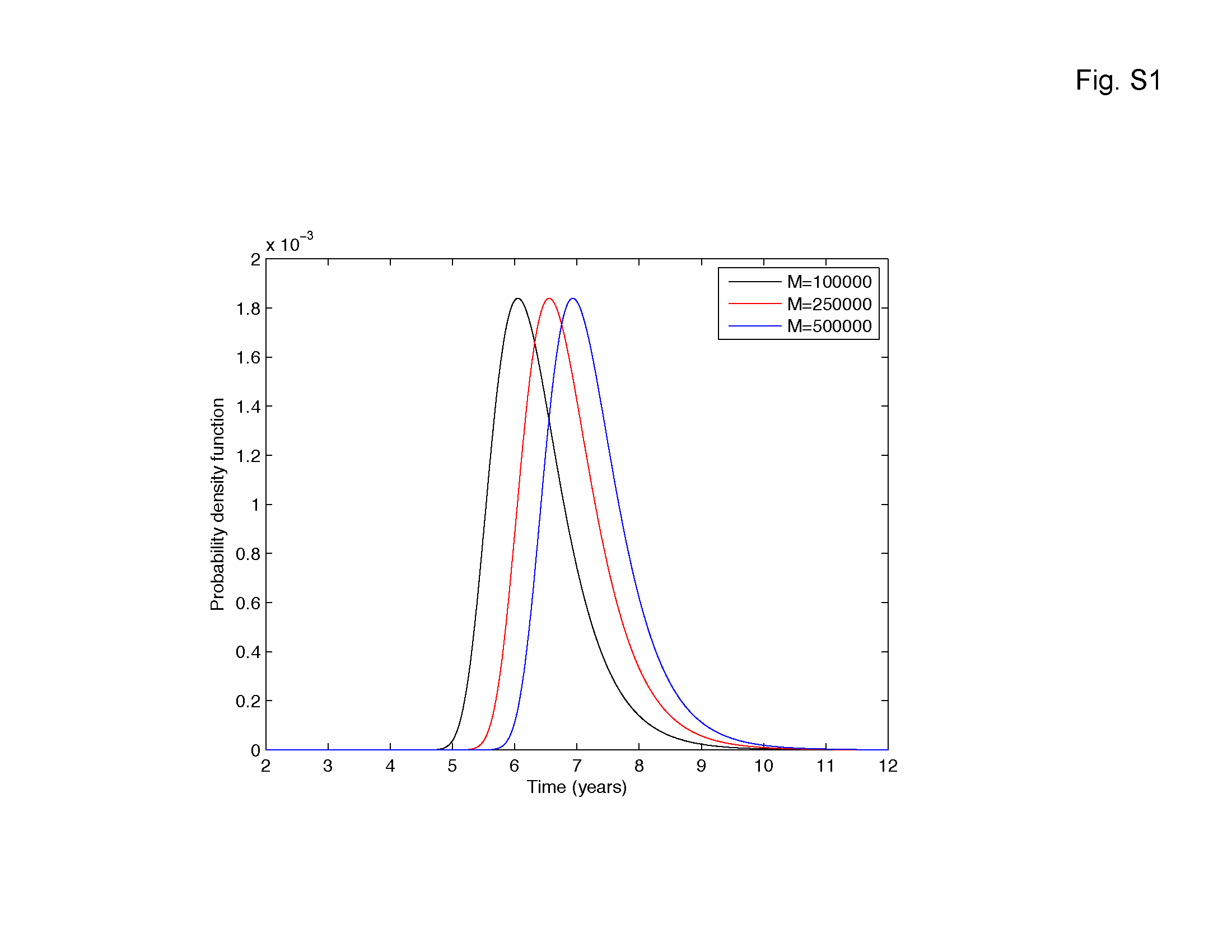

Supplement: Figure S1 — Time of detection of disease. The figure shows the probability density function of the detection time, or the time that the CML stem cell population hits size M, for M = 100, 000, 250, 000 and 500, 000. Since the mutant population does not represent a significant portion of the population at detection, this distribution is closely approximated by considering the time at which the number of drug-sensitive CML stem cells reaches M. (TIFF) [file pone.0027682.s001.tif]

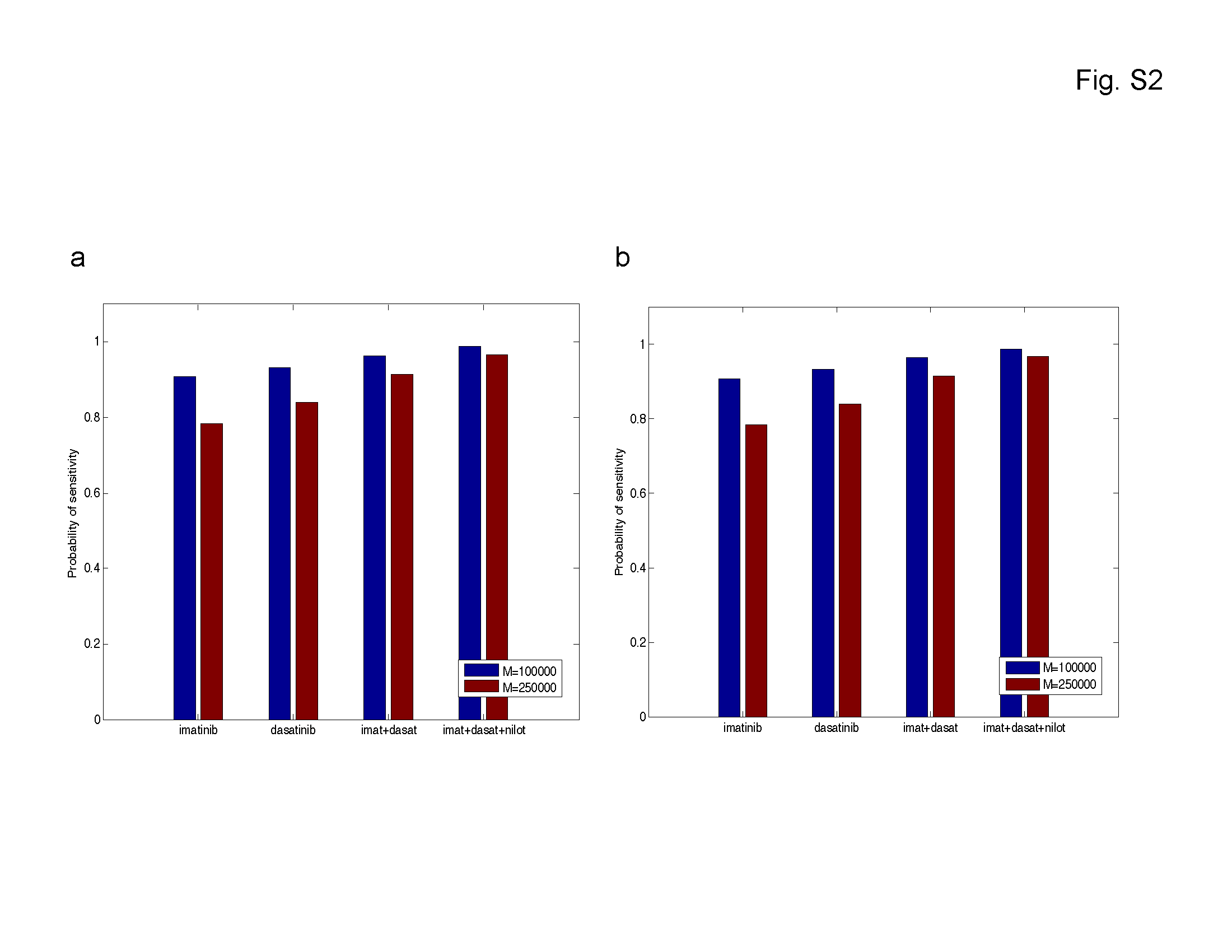

Supplement: Figure S2 — Robustness to growth rate perturbations. a) Probability of sensitivity to mono- and combination therapies when the resistant mutant birth rates are perturbed by a multiplicative random factor (1+0.2·U [−1, 1]), for one representative sample (see Table S1 for comprehensive robustness statistics). b) Probability of sensitivity to mono- and combination therapies when the fitness differences between mutants are attributed to variation in death rates instead of birth rates. In both panels, probabilities are shown for detection sizes of 100,000 and 250,000 cells. (TIFF) [file pone.0027682.s002.tif]

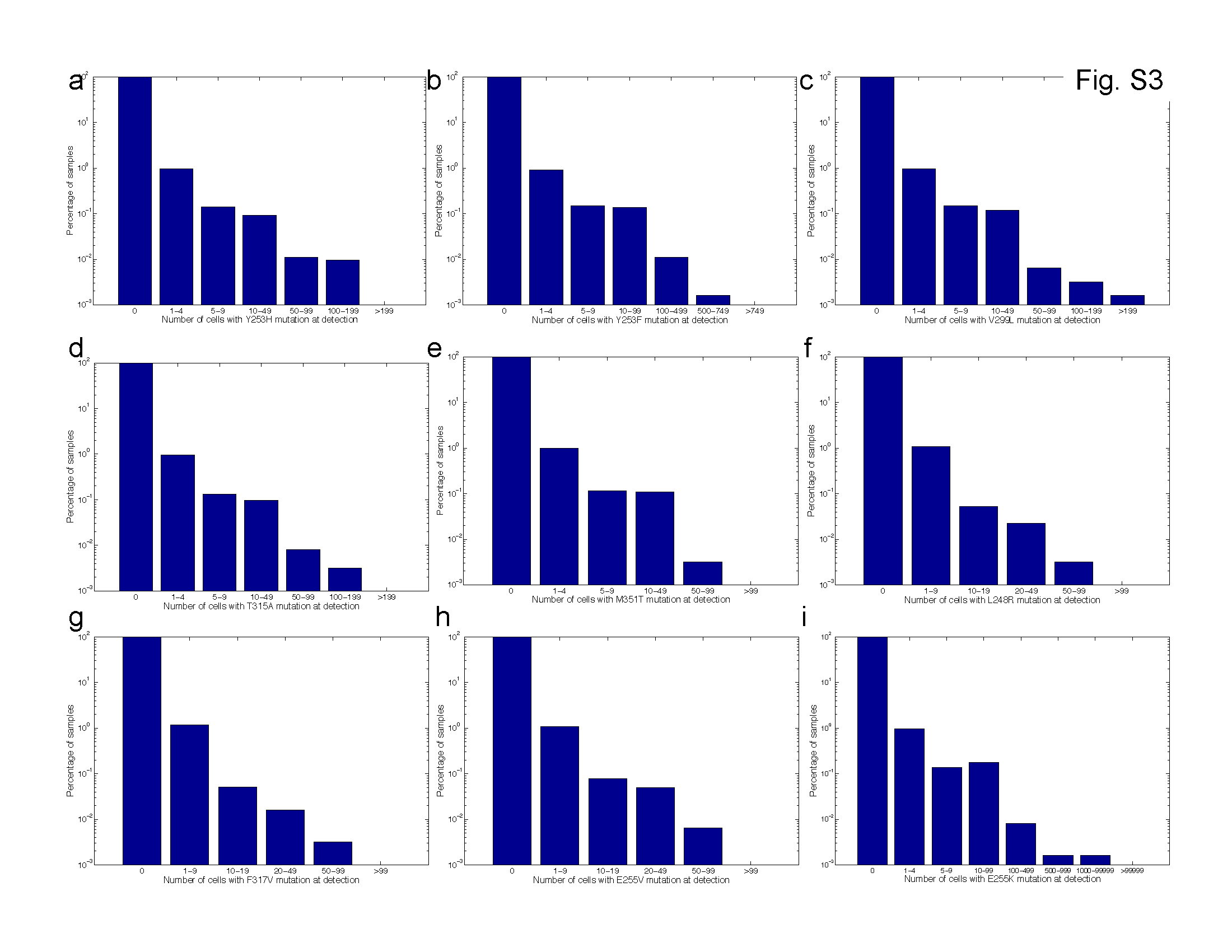

Supplement: Figure S3 — The frequency of CML resistance mutations at diagnosis. The figure shows the distribution of the number of Y253H-positive (a), Y253F-positive (b), V299L- positive (c), T315A-positive (d), M351T-positive (e), L248R-positive (f), F317V-positive (g), E255V-positive (h), and E255K-positive (i) cells in the population at detection time. Parameters are M = 100, 000 and u = 10−7, and simulations are run for 100,000 samples. (TIFF) [file pone.0027682.s003.tif]
