# Supplementary material for: Fitness Conferred by BCR-ABL Kinase Domain Mutations Determines the Risk of Pre-Existing Resistance in Chronic Myeloid Leukemia
Source: PLoS One. 2011 Nov 28;6(11):e27682. doi: 10.1371/journal.pone.0027682 (PMC3225363; doi:10.1371/journal.pone.0027682)
Supplement: Table S1 — Robustness properties. (PDF) [file pone.0027682.s004.pdf]

TABLE S1: ROBUSTNESS PROPERTIES

	Probability (unperturbed rates)	Max observed	Min observed
I (M1)	0.9053	0.9061	0.9046
D (M1)	0.9287	0.9294	0.9281
I+D (M1)	0.9636	0.9640	0.9632
I +D+N(M1)	0.9874	0.9876	0.9872
I (M2)	0.7798	0.7816	0.7784
D (M2)	0.8312	0.8328	0.8297
I+D (M2)	0.9115	0.9125	0.9106
I +D+N(M2)	0.9687	0.9693	0.9682
