# Supplementary material for: Fitness Conferred by BCR-ABL Kinase Domain Mutations Determines the Risk of Pre-Existing Resistance in Chronic Myeloid Leukemia
Source: PLoS One. 2011 Nov 28;6(11):e27682. doi: 10.1371/journal.pone.0027682 (PMC3225363; doi:10.1371/journal.pone.0027682)
Supplement: Material S1 — Supplementary Material. (PDF) [file pone.0027682.s005.pdf]

SUPPLEMENTARY MATERIAL: FITNESS CONFERRED BY BCR-ABL KINASE DOMAIN MUTATIONS DETERMINES THE RISK OF PRE-EXISTING RESISTANCE IN CHRONIC MYELOID LEUKEMIA

KEVIN LEDER, JASMINE FOO, BRIAN J. SKAGGS, MERCEDES GORRE, CHARLES L. SAWYERS, AND FRANZISKA MICHOR

1. Robustness of results to in vivo growth rate perturbation

We perturbed the growth rate estimates for the resistant mutants by the multiplicative random factor $(1 + 0.2 \cdot U[-1, 1])$ representing a 20% noise level, and performed 100 randomized trials. Table S1 shows the probability of sensitivity to imatinib (I), dasatinib (D), imatinib + dasatinib (I+D), and imatinib + dasatinib + nilotinib (I+D+N) for the baseline unperturbed growth rates, as well as the minimum and maximum analogous probabilities observed in the 100 trials with randomized perturbed growth rates (Table S1). The notation (M1) denotes the estimates for a detection size of $100,000$ cells, and (M2) denotes estimates for a detection size of $250,000$ cells.

2. Analysis of the mathematical model

In the following section we first provide derivations for the mode, median and mean of the detection time. We then present derivations for the distribution of the number of distinct resistant types, the population size of each resistant type, and the probability of resistance as a function of time and at the random time of detection.

2.1. **Detection Time.** Let us first investigate the behavior of the process at the time of disease diagnosis. The detection time is defined as the first time that the leukemic stem cell population hits size M. Recall that a good approximation for the BCR-ABL^{p210} cell population at time t is $Ve^{\lambda_0 t}$, where V has density $f_V(x) = (\lambda_0/a_0)e^{-x\lambda_0/a_0}$. In the previous formula, λ_0 represents the net growth rate of the BCR-ABL^{p210} cells, and a_0 represents their birth rate. This approximation is based on the scaled limit of a binary Markov branching process conditioned on never going extinct. Therefore, the distribution of the detection of the disease can be modeled using the distribution of the first time the process $Ve^{\lambda_0 t}$ hits size M, which is denoted by τ_M. This random variable τ_M has density

$$\rho_{\tau_M}(t) = \frac{\lambda_0^2 M}{a_0} \exp[-(\lambda_0 M/a_0)\exp(-\lambda_0 t)]\exp(-\lambda_0 t) \tag{1}$$

and cdf

$$F_{\tau_M}(t) = \exp[-e^{-\lambda t} M\lambda_0/a].$$

The mode of this distribution is given by

$$m_0 = \frac{1}{\lambda_0}\log\left(\frac{\lambda_0 M}{a_0}\right),$$

its median by

$$\begin{aligned} t_{1/2} &= -\frac{1}{\lambda_0}\log\left[-\frac{a_0}{M\lambda_0}\log\left[\frac{1}{2}+e^{-M\lambda_0/a_0}\right]\right] \\ &\approx \frac{1}{\lambda_0}\log\left(\frac{M\lambda_0}{a_0}\right) - \frac{1}{\lambda_0}\log\log 2, \end{aligned}$$

and if we define γ to be the Euler-Mascheroni constant, then its mean is given by

$$\begin{aligned} E[\tau_M] &= \frac{1}{\lambda_0}\gamma + \frac{1}{\lambda_0}\log\left(\frac{M\lambda_0}{a_0}\right) + \frac{1}{\lambda_0}\int_{\frac{M\lambda_0}{a_0}}^{\infty}\frac{e^{-z}}{z}dz \\ &\approx \frac{1}{\lambda_0}\log\left(\frac{M\lambda_0}{a_0}\right) + \frac{1}{\lambda_0}\gamma. \end{aligned}$$

The approximations above follow by recalling that M is $O(10^5)$. The mode is found by maximizing the density, and the median is found by solving $F_{\tau_M}(t_{1/2}) = 1/2$. The expression for the mean is found as follows:

$$\begin{aligned} E[\tau_M] &= \frac{\lambda_0^2 M}{a_0}\int_0^{\infty} t e^{-\lambda_0 t}\exp\left(-\frac{\lambda_0 M}{a_0}e^{-\lambda_0 t}\right)dt = -\frac{1}{\lambda_0}\int_0^{\frac{\lambda_0 M}{a_0}} e^{-z}\log\left(\frac{a_0 z}{\lambda_0 M}\right)dz \\ &= -\frac{1}{\lambda_0}\int_0^{\infty} e^{-z}\log\left(\frac{a_0 z}{M\lambda_0}\right)dz + \frac{1}{\lambda_0}\int_{\frac{M\lambda_0}{a_0}}^{\infty} e^{-z}\log\left(\frac{a_0 z}{M\lambda_0}\right)dz \\ &= -\frac{1}{\lambda_0}\int_0^{\infty} e^{-z}\log\left(\frac{a_0 z}{M\lambda_0}\right)dz + \frac{1}{\lambda_0}\int_{\frac{M\lambda_0}{a_0}}^{\infty}\frac{e^{-z}}{z}dz \\ &= \frac{1}{\lambda_0}\log\left(\frac{M\lambda_0}{a_0}\right) - \frac{1}{\lambda_0}\int_0^{\infty} e^{-z}\log z dz + \frac{1}{\lambda_0}\int_{\frac{M\lambda_0}{a_0}}^{\infty}\frac{e^{-z}}{z}dz, \end{aligned}$$

where the first equality follows from the substitution $z = (\lambda_0 M/a_0)e^{-\lambda_0 t}$, and the penultimate equality follows from integration by parts. The final formula for $E[\tau_M]$ follows by recalling the identity

$$\gamma = -\int_0^{\infty} e^{-z}\log z dz,$$

and making the approximation (which is certainly valid for $M \geq 10^5$)

$$\frac{1}{\lambda_0}\int_{\frac{M\lambda_0}{a_0}}^{\infty}\frac{e^{-z}}{z}dz \approx 0.$$

2.2. **Number of distinct types and probability of resistance at time t.** Let us first investigate the number of mutations that have emerged before a deterministic time t.

2.2.1. *Number of mutations by time t.* Let $N(t)$ be the total number of mutations that have arisen until time t, $N_i(t)$ be the total number of type i mutations, and u the per base pair mutation rate of BCR-ABL^{p210}-positive cells. Mutations arrive as a Poisson process with intensity at time $s \le t$ given by $W(s)ua_0n$ and mutations to type i have intensity $a_0uW(s)$. Each mutation occurs with the same intensity. If we condition on the random variable V, we have

$$\begin{aligned} P(N(t)=k|V) &= \frac{1}{k!}\left(na_0uV\int_0^t e^{\lambda_0 s}ds\right)^k \exp\left(-na_0uV\int_0^t e^{\lambda_0 s}ds\right) \\ &= \left(\frac{na_0u\left(e^{\lambda_0 t}-1\right)}{\lambda_0}\right)^k \frac{V^k}{k!}\exp\left[-nV\left(\frac{a_0u\left(e^{\lambda_0 t}-1\right)}{\lambda_0}\right)\right]. \end{aligned}$$

Then, if we integrate out the distribution of V, we see that

$$P(N(t)=k) = \frac{\lambda_0^2}{\lambda_0^2 + na_0^2u(e^{\lambda_0 t}-1)}\left(\frac{na_0^2u(e^{\lambda_0 t}-1)}{\lambda_0^2+na_0^2u(e^{\lambda_0 t}-1)}\right)^k,$$

for k a non-negative integer. It follows immediately that

$$\begin{aligned} E[N(t)|V] &= \frac{na_0uV\left(e^{\lambda_0 t}-1\right)}{\lambda_0} \\ E[N(t)] &= nu\left(\frac{a_0}{\lambda_0}\right)^2\left(e^{\lambda_0 t}-1\right), \end{aligned}$$

and

$$E[N_i(t)] = E[N(t)]/n,$$

for $1 \le i \le n$. This quantity represents the gross number of mutations to arrive by time t and therefore also counts mutations whose offspring eventually die out. In order to adjust for mutations that might die out we simply consider the arrival process of mutations whose offspring are still viable at time t. For a binary branching process with birth rate a, death rate b and net growth rate $\lambda = a - b$, define

$$p(t,a,b) = \frac{\lambda}{a - be^{-\lambda t}}. \tag{2}$$

This is the probability that the branching process has not gone extinct by time t. Therefore if we let $\bar{N}_i(t)$ be the number of mutations that have occurred by time t and have viable offspring at time t, then this process is a Poisson process with intensity at time $s \le t$ given by $a_0uW(s)p(t-s,a_i,b_0)$. Therefore, if we define

$$J_i(t) = \int_0^t e^{\lambda_0 s}p(t-s,a_i,b_0)ds = \lambda_i\int_0^t \frac{e^{\lambda_0 s}}{a_i - b_0e^{-\lambda_i(t-s)}}ds,$$

we see that for each $1 \leq i \leq n$,

$$P\left(\bar{N}_i(t) = k|V\right) = \frac{V^k\left(a_0 u J_i(t)\right)^k}{k!}\exp\left(-a_0 u V J_i(t)\right) \tag{3}$$
$$P\left(\bar{N}_i(t) = k\right) = \frac{\lambda_0}{\lambda_0 + a_0^2 u J_i(t)}\left(\frac{a_0^2 u J_i(t)}{\lambda_0 + a_0^2 u J_i(t)}\right)^k,$$

for n a non-negative integer.

2.2.2. *Number of distinct types.* A quantity of interest is the number of distinct viable mutant populations at time t; that is, from our list of n possible mutations, how many of them have viable offspring at time t. Let us denote this quantity by $N_d(t)$. We first consider $P(N_d(t) = k)$ for $1 \leq k \leq n$. For each $1 \leq k \leq n$, define

$$\mathcal{N}_k = \{A \subset \{1, \ldots, n\} : |A| = k\}.$$

Then the probability mass function for the number of distinct types present at time t has the form

$$P(N_d(t) = k|V) = \sum_{A \in \mathcal{N}_k} \exp\left(-a_0 u V \sum_{i \notin A} J_i(t)\right)\left(\prod_{i \in A}\left[1 - \exp\left(-a_0 u V J_i(t)\right)\right]\right) \tag{4}$$
$$P(N_d(t) = k) = \sum_{A \in \mathcal{N}_k}\left(\prod_{j \notin A} P\left(\bar{N}_j(t) = 0\right)\right)\left(\prod_{j \in A} P\left(\bar{N}_j(t) > 0\right)\right).$$

There is a simple representation formula for $N_d(t)$, given by

$$N_d(t) = \sum_{i=1}^{n} I(\bar{N}_i(t) = 0),$$

where $I(\cdot)$ represents an indicator function. Based on this, it follows that the expected number of distinct types at time t is

$$E[N_d(t)|V] = \sum_{i=1}^{n} P(\bar{N}_i(t) > 0|V) = \sum_{i=1}^{n}\left(1 - \exp\left(-a_0 u V J_i(t)\right)\right) \tag{5}$$
$$E[N_d(t)] = \sum_{i=1}^{n} P(\bar{N}_i(t) > 0) = \sum_{i=1}^{n} \frac{a_0^2 u J_i(t)}{\lambda_0 + a_0^2 u J_i(t)}.$$

It is also possible to obtain the following expression for the variance of N_d:

$$\mathrm{Var}[N_d(t)] = \sum_{i=1}^{n}\sum_{j=1}^{n}\left(P\left(\bar{N}_i(t) > 0, \bar{N}_j(t) > 0\right) - P\left(\bar{N}_i(t) > 0\right)P\left(\bar{N}_j(t) > 0\right)\right).$$

2.2.3. *Probability of resistance.* We can also evaluate the probability that there are no mutations present in the population at time t:

$$P\left(\mathcal{M}(t)=0|V\right)=\exp\left(-a_0uV\sum_{i=1}^{n}J_i(t)\right) \tag{6}$$

$$P(\mathcal{M}(t)=0)=\prod_{i=1}^{n}P(\bar{N}_i(t)=0)=\prod_{i=1}^{n}\frac{\lambda_0}{\lambda_0+a_0^2uJ_i(t)}.$$

The probability of any particular resistant type, $1\leq i\leq n$, existing at time t can be calculated from equation (3) as

$$P\left(\mathcal{M}_i(t)>0|V\right)=1-\exp\left(-a_0uVJ_i(t)\right)$$

$$P\left(\mathcal{M}_i(t)>0\right)=1-\frac{\lambda_0}{\lambda_0+a_0^2uJ_i(t)}.$$

2.3. **Mutant population at time t.** Let us now determine the expected total number of resistant cells at detection time and the expected number of resistant cells for each specific mutation. These expected values are obtained by investigating the distribution of the times at which the mutations occur. The arrival times of an inhomogeneous Poisson process can be characterized as follows: given that $N(t)=n$, the times when the mutations occur in $[0,t]$, $\tau_1(t),\ldots,\tau_n(t)$ are distributed as i.i.d samples from the density

$$f_t(s)=\frac{\lambda_0e^{\lambda_0s}}{e^{\lambda_0t}-1}.$$

One immediate consequence of this result is that mutations are much more likely to occur immediately prior to detection.

It is possible to represent the population of mutants at time t by the following formula:

$$\mathcal{M}(t)=\sum_{j=1}^{N(t)}Z_j(t-\tau_j(t)), \tag{7}$$

where Z_j represents the jth birth-death process created via mutation of the original W process.

In order for our representation to be useful, it is necessary to study the mean and variance of the random variables $Z_j(t-\tau_j(t),y)$. To clarify, this random variable represents the size at time t of one of the mutant birth-death processes created in the interval $[0,t]$. Therefore, by conditioning on the value of $\tau_j(t)$ and the type of mutation that occurred, and recalling the expected value of a birth-death process at time t, we can write the mean as follows:

$$\mu(t)\doteq EZ_1(t-\tau_1(t))=\frac{\lambda_0}{n(e^{\lambda_0t}-1)}\sum_{i=1}^{n}\int_0^t\exp\left[(t-s)(\lambda_i)\right]e^{\lambda_0s}ds$$

$$=\frac{\lambda_0}{n(e^{\lambda_0t}-1)}\sum_{i=1}^{n}\frac{e^{\lambda_0t}-e^{\lambda_it}}{\lambda_0-\lambda_i},$$

and similarly we can consider $\mu_i(t) = E[Z_1^i(t - \tau_1(t))]$, where Z_1^i only consider mutations of type i,

$$\mu_i(t) = \frac{\lambda_0}{(e^{\lambda_0 t} - 1)} \frac{e^{\lambda_0 t} - e^{\lambda_i t}}{\lambda_0 - \lambda_i}.$$

Based on this we can calculate the following:

$$E\left[\mathcal{M}(t)\right] = E[N(t)]\mu(t) = \frac{ua_0}{\lambda_0} \sum_{i=1}^{n} \frac{e^{\lambda_0 t} - e^{\lambda_i t}}{\lambda_0 - \lambda_i}, \tag{8}$$

and, by a similar calculation,

$$E\left[\mathcal{M}_i(t)\right] = \left(\frac{ua_0}{\lambda_0}\right) \frac{e^{\lambda_0 t} - e^{\lambda_i t}}{\lambda_0 - \lambda_i}. \tag{9}$$

2.4. **Mutant population at detection time.** In this section, we combine the results of the previous two sections and analyze the quantities in sections 2.2 with the results in 2.1.

We first consider the probability of having no mutations at detection time. Using the approximation τ_M for the detection time, the observation $\tau_M = t \Leftrightarrow V = Me^{-\lambda_0 t}$, and the formula from (6), we obtain

$$\begin{aligned} P\left(\mathcal{M}(\tau_M) = 0\right) &= \int_0^\infty P(\mathcal{M}(t) = 0 | \tau_M = t) \rho_{\tau_M}(t) dt \\ &= \int_0^\infty P(\mathcal{M}(t) = 0 | V = Me^{-\lambda_0 t}) \rho_{\tau_M}(t) dt \\ &= \int_0^\infty \exp\left(-a_0 u M e^{-\lambda_0 t} \sum_{i=1}^{n} J_i(t)\right) \rho_{\tau_M}(t) dt. \end{aligned}$$

Plugging in the definition of ρ_{τ_M} gives

$$P\left(\mathcal{M}(\tau_M) = 0\right) = \frac{\lambda_0^2 M}{a_0} \int_0^\infty e^{-\lambda_0 t} \exp\left[-Me^{-\lambda_0 t}\left(\frac{\lambda_0}{a_0} + a_0 u \sum_{i=1}^{n} J_i(t)\right)\right] dt.$$

Then making the change of variables $s = Me^{-\lambda_0 t}$ gives

$$P\left(\mathcal{M}(\tau_M) = 0\right) = \frac{\lambda_0}{a_0} \int_0^M \exp\left[-s\left(\frac{\lambda_0}{a_0} + a_0 u \sum_{i=1}^{n} J_i\left(-\frac{1}{\lambda_0} \log \frac{s}{M}\right)\right)\right] ds.$$

Note that

$$\begin{aligned} J_i(t) &= \lambda_i \int_0^t \frac{e^{\lambda_0 s}}{a_i - b_0 e^{-\lambda_i (t-s)}} ds = \lambda_i e^{\lambda_0 t} \int_0^t \frac{e^{-\lambda_0 (t-s)}}{a_i - b_0 e^{-\lambda_i (t-s)}} ds \\ &= \lambda_i e^{\lambda_0 t} \int_0^t \frac{e^{-\lambda_0 s}}{a_i - b_0 e^{-\lambda_i s}} ds, \end{aligned}$$

and therefore

$$J_i\left(\frac{1}{\lambda_0} \log \frac{M}{s}\right) = \frac{\lambda_i M}{s} \int_0^{\frac{1}{\lambda_0} \log \frac{M}{s}} \frac{e^{-\lambda_0 r}}{a_i - b_0 e^{-\lambda_i r}} dr.$$

The result in the previous display can be plugged into the previous formula for $P(\mathcal{M}(\tau_M) = 0)$ to get

$$P(\mathcal{M}(\tau_M) = 0) = \frac{\lambda_0}{a_0} \int_0^M e^{-s\lambda_0/a_0} \exp\left[-a_0 u \lambda_i M \sum_{i=1}^n \int_0^{\frac{1}{\lambda_0}\log\frac{M}{s}} \frac{e^{-\lambda_0 r}}{a_i - b_0 e^{-\lambda_i r}} dr\right] ds. \tag{10}$$

One can also easily calculate the probability that there are no type i cells for $1 \le i \le n$ at detection,

$$P(\mathcal{M}_i(\tau_M) = 0) = \frac{\lambda_0}{a_0} \int_0^M e^{-s\lambda_0/a_0} \exp\left[-a_0 u \lambda_i M \int_0^{\frac{1}{\lambda_0}\log\frac{M}{s}} \frac{e^{-\lambda_0 r}}{a_i - b_0 e^{-\lambda_i r}} dr\right] ds. \tag{11}$$

Using equation (5), we can look at the expected number of distinct mutants at detection time

$$E[N_d(\tau_M)] = \int_0^\infty \rho_{\tau_M}(t) \sum_{i=1}^n \left[1 - \exp(-a_0 u e^{-\lambda_0 t} M J_i(t))\right] dt.$$

Following the same procedure as in the derivation of the formula for $P(\mathcal{M}(\tau_M) = 0)$, we arrive at the following

$$\begin{aligned} E[N_d(\tau_M)] &= n - \frac{\lambda_0}{a_0} \sum_{i=1}^n \int_0^M \exp\left[-s\left(\frac{\lambda_0}{a_0} + a_0 u J_i\left(-\frac{1}{\lambda_0}\log\frac{s}{M}\right)\right)\right] ds \\ &= n - \frac{\lambda_0}{a_0} \sum_{i=1}^n \int_0^M e^{-s\lambda_0/a_0} \exp\left[-a_0 u \lambda_i M \int_0^{\frac{1}{\lambda_0}\log\frac{M}{s}} \frac{e^{-\lambda_0 r}}{a_i - b_0 e^{-\lambda_i r}} dr\right] ds. \end{aligned} \tag{12}$$

These results can be generalized, and it is possible to look at the distribution for the number of distinct types present at detection time

$$\begin{aligned} P(N_d(\tau_M) = k) &= \int_0^\infty P(N_d(t) = k | V = Me^{-\lambda_0 t}) \rho_{\tau_M}(t) dt \\ &= \frac{\lambda_0^2 M}{a_0} \int_0^\infty P(N_d(t) = k | V = Me^{-\lambda_0 t}) e^{-\lambda_0 t} \exp\left[-\left(\frac{\lambda_0 M}{a_0}\right) e^{-\lambda_0 t}\right] dt. \end{aligned}$$

If we plug in the definition of $P(N_d(t) = k|V)$ from (4), make the change of variable $s = Me^{-\lambda_0 t}$, and then the replacement

$$J_i\left(\frac{1}{\lambda_0}\log\frac{M}{s}\right) = \frac{\lambda_i M}{s} \int_0^{\frac{1}{\lambda_0}\log\frac{M}{s}} \frac{e^{-\lambda_0 r}}{a_i - b_0 e^{-\lambda_i r}} dr,$$

we get

$$\begin{aligned} P(N_d(\tau_M) = k) = \frac{\lambda_0}{a_0} \sum_{A \in \mathcal{N}_k} \int_0^M &\exp\left[-s\lambda_0/a_0 + a_0 u M \sum_{i \notin A} \lambda_i \int_0^{\frac{1}{\lambda_0}\log\frac{M}{s}} \frac{e^{-\lambda_0 r}}{a_i - b_0 e^{-\lambda_i r}} dr\right] \\ &\times \prod_{i \in A} \left[1 - \exp\left(-a_0 u M \lambda_i \int_0^{\frac{1}{\lambda_0}\log\frac{M}{s}} \frac{e^{-\lambda_0 r}}{a_i - b_0 e^{-\lambda_i r}} dr\right)\right] ds. \end{aligned} \tag{13}$$

It is also possible to look at the expected population level of each mutant type at detection. In particular we have the following by plugging in the definition of $E[N(t)|V]$, $\mu(t)$ and $\rho_{\tau_M}(t)$ respectively,

$$\begin{aligned} E[\mathcal{M}_i(\tau_M)] &= \int_0^\infty \mu_i(t) E[N_i(t)|V = Me^{-\lambda_0 t}]\rho_{\tau_M}(t)dt \\ &= \frac{a_0 u M}{\lambda}\int_0^\infty \mu_i(t)(1-e^{-\lambda_0 t})\rho_{\tau_M}(t)dt \\ &= a_0 u M \int_0^\infty \rho_{\tau_M}(t)\frac{1-e^{-\lambda_0 t}}{e^{\lambda_0 t}-1}\frac{e^{\lambda_0 t}-e^{\lambda_i t}}{\lambda_0-\lambda_i}dt \\ &= u\lambda_0^2 M^2 \int_0^\infty e^{-\lambda_0 t}\exp\left[-\left(\frac{\lambda_0 M}{a_0}\right)e^{-\lambda_0 t}\right]\frac{1-e^{(\lambda_i-\lambda_0)t}}{\lambda_0-\lambda_i}dt \\ &= u\lambda_0 M^2 \int_0^1 \frac{(1-y^{1-\lambda_i/\lambda_0})e^{-y\lambda_0 M/a_0}}{\lambda_0-\lambda_i}dy, \end{aligned} \tag{14}$$

where the final equality follows from a change of variable $y = e^{-\lambda_0 t}$. Note that the above formula is only valid for mutant types with birth rate less than or equal to the birth rate of the sensitive cells, i.e., neutral or disadvantageous mutants.

3. Monte Carlo simulation

Suppose one is interested in estimating $E[f(\mathcal{M}(\tau_M))]$ for some function f, and it is possible to simulate K (where K is a large integer) independent copies of $\{\mathcal{M}^{(i)}(\tau_M)\}_{i=1}^K$. Then, from the law of large numbers, we obtain that

$$E[f(\mathcal{M}(\tau_M))] \approx \frac{1}{K}\sum_{i=1}^K f\left(\mathcal{M}^{(i)}(\tau_M)\right).$$

From the central limit theorem, we get that this approximation has error

$$\sqrt{\frac{Var[f(\mathcal{M}(\tau_M))]}{K}}$$

and therefore its relative error is

$$\frac{1}{\sqrt{K}}\frac{\sqrt{Var[f(\mathcal{M}(\tau_M))]}}{E[f(\mathcal{M}(\tau_M))]}.$$

Therefore, assuming that as $M \to \infty$, $\sqrt{Var[f(\mathcal{M}(\tau_M))]}/(E[f(\mathcal{M}(\tau_M))])$ stays bounded, it is possible to use the Monte Carlo method to get good approximations to functions of the mutant population at detection.

4. Figures and Tables

.

Figure S1. Time of detection of disease. The figure shows the probability density function of the detection time, or the time that the CML stem cell population hits size M, for $M = 100,000, 250,000$ and $500,000$. Since the mutant population does not represent a significant portion of the population at detection, this distribution is closely approximated by considering the time at which the number of drug-sensitive CML stem cells reaches M.

Figure S2. Robustness to growth rate perturbations. a) Probability of sensitivity to mono- and combination therapies when the resistant mutant birth rates are perturbed by a multiplicative random factor $(1 + 0.2 \cdot U[-1, 1])$, for one representative sample (see Table S1 for comprehensive robustness statistics). b) Probability of sensitivity to mono- and combination therapies when the fitness differences between mutants are attributed to variation in death rates instead of birth rates. In both panels, probabilities are shown for detection sizes of 100,000 and 250,000 cells.

Figure S3. The frequency of CML resistance mutations at diagnosis. The figure shows the distribution of the number of Y253H-positive (a), Y253F-positive (b), V299L-positive (c), T315A-positive (d), M351T-positive (e), L248R-positive (f), F317V-positive (g), E255V-positive (h), and E255K-positive (i) cells in the population at detection time. Parameters are $M = 100,000$ and $u = 10^{-7}$, and simulations are run for 100,000 samples.

Table S1. Robustness properties.
